# Layer Thickness Effects on the Ageing Performance and Mould Resistance of Polyurethane-Coated Beech Wood

**DOI:** 10.3390/polym18091145

**Published:** 2026-05-06

**Authors:** Gabriela Slabejová, Zuzana Vidholdová

**Affiliations:** 1 Department of Furniture and Wood Products, Faculty of Wood Sciences and Technology, Technical University in Zvolen, T. G. Masaryka 24, 96001 Zvolen, Slovakia; slabejova@tuzvo.sk; 2 Department of Wood Technology, Faculty of Wood Sciences and Technology, Technical University in Zvolen, T. G. Masaryka 24, 96001 Zvolen, Slovakia

**Keywords:** beech wood, colour, gloss, moulds, polyurethane

## Abstract

The paper deals with the optical stability and biological resistance of solvent-based polyurethane and water-based polyurethane–acrylate finishes intended for interior use, applied on beech wood with mature wood and false heartwood. Optical stability was assessed through colour and gloss measurements. The surface finishes were applied in one (I), two (II), and three (III) layers. Natural ageing was carried out under indoor conditions with exposure to natural daylight behind a glass window. Colour and gloss measurements were performed after 30, 150, and 300 days of exposure. The development of mould growth activity (GAM) during 21-day exposure was assessed on untreated and coated beech wood with different coating thicknesses. A significant colour difference in the solvent-based polyurethane finish on both mature wood and false heartwood occurred after 30 days of natural ageing. The colour difference in the water-based polyurethane-acrylate finish on both mature wood and false heartwood increased with exposure time. The solvent-based polyurethane finish with three layers applied to false heartwood showed reduced colour difference (by 62.5%). Natural ageing under interior conditions did not significantly affect the gloss of the matte surface for either finish on both mature wood and false heartwood. The degree of mould growth decreased with an increasing number of coating layers for both polyurethane and polyurethane-acrylate finishes.

## 1. Introduction

Polyurethane coatings are widely used for the protection and aesthetic enhancement of interior wood products due to their favourable mechanical properties, transparency, and chemical resistance. With the enhancement of people’s environmental awareness, waterborne polyurethane paint is used, with its advantages of low release of volatile organic compounds (VOCs) [1,2,3,4]. Transparent polyurethane systems (solvent-based and water-based) are particularly valued for maintaining the natural appearance of wood while providing a durable surface barrier [5]. In indoor environments, coated wooden elements are exposed to gradual ageing processes [6], which can affect both their optical properties and resistance to microbial colonisation [7].

The ageing of polyurethane coatings indoors is primarily governed by photo-oxidative and thermochemical reactions. Exposure to ultraviolet (UV) and visible radiation initiates chain scission in polymeric chains, oxidation of urethane linkages, and the formation of chromophore groups, leading to colour changes and gloss reduction [6,8,9,10,11]. Studies [5,12,13,14,15] have also documented material degradation due to outdoor and indoor ageing of wood coatings, with clear evidence of colour shifts and surface morphology changes. Although accelerated ageing tests are often used to simulate long-term exposure, discrepancies between natural and laboratory conditions remain a critical issue for realistic durability assessment. In addition to physical-chemical degradation, coated wood surfaces may be susceptible to microbial colonisation, particularly moulds, when appropriate microclimatic conditions exist. Mould growth is influenced by surface moisture, nutrient availability, and temperature, and certain fungi, including *Aspergillus*, *Penicillium*, and *Cladosporium*, can degrade polyurethane coatings [8,16,17]. Ageing-induced microcracks, increased surface roughness, and changes in surface polarity may facilitate spore adhesion and fungal growth, indicating that biological resistance cannot be considered independent of coating ageing [18].

One critical parameter affecting coating performance is layer thickness, which influences UV penetration, oxygen diffusion, internal stress distribution, and moisture transport properties [19]. Thicker films can delay photochemical degradation by acting as an effective barrier; however, they may also alter vapour permeability and interfacial moisture accumulation, potentially increasing susceptibility to colonisation with rot-fungi [17]. The coatings with different thickness and resin composition [1,5,20,21,22,23] exhibit varied mechanical properties, resistance to both UV-induced changes and biological attack [14]. Despite its practical importance, the thickness-dependent ageing behaviour of polyurethane coatings on wood substrates has not been comprehensively evaluated.

Moreover, the wood substrate itself plays a decisive role in coating durability. Beech wood (*Fagus sylvatica* L.) is widely used in furniture and interior applications [24,25], but notable variability exists between mature wood and red heartwood [26,27,28,29]. The occurrence of false heartwood in beech wood is a common defect that is encountered in practice. Nevertheless, the mechanical properties of a false heartwood of beech wood without rot do not differ from mature wood [29]. It is appropriate to consider the idea of using beech wood with a false heartwood for the production of interior products. One option is to use the colour contrast between the false heartwood and mature wood, which brings a new aesthetic element to the product, or to unify the colour by treating the wood [11,27].

Differences in chemical composition, extractive content, and colour characteristics may affect light absorption, migration of low-molecular-weight compounds, and interfacial interactions with coatings, thereby influencing both optical stability and mould susceptibility.

Although numerous studies have investigated polyurethane degradation or mould resistance separately, limited attention has been given to their simultaneous evaluation in relation to coating thickness and wood substrate type under indoor conditions. A comprehensive assessment integrating optical and biological indicators is necessary to better understand the service performance of coated wood products [30].

Therefore, the aim of this study was to evaluate the influence of layer thickness on the ageing performance of polyurethane coatings applied to radial surfaces of beech wood in the zone of mature and false heartwood under natural interior conditions. Radial surfaces of beech wood are often found on veneered furniture, but also on solid wood furniture. Optical stability was assessed through colour and gloss measurements, while biological resistance was determined via mould growth evaluation. This study seeks to determine whether coating thickness is a governing factor in maintaining surface stability and to identify substrate-dependent differences in ageing response, providing insights for the optimisation of durable interior wood protection strategies.

## 2. Materials and Methods

### 2.1. Material and Surface Treatment

Beech (*Fagus sylvatica* L.) samples with a zone of false heartwood and mature wood with radial top surface were used with dimensions 100 mm × 100 mm × 3 mm (exposure in natural ageing) and 50 mm × 50 mm × 3 mm (resistance to mould fungi). The average density of samples was 676 kg/m^3,^ and the average moisture content of air-conditioned samples was 8 ± 2%. All surfaces of testing samples were ground by sandpaper (gradually with grain size numbers P60, P80, P120, and P150) using the belt grinder machine—Festool ETS 120 abrasive Rubin 2, with a diameter of 125 mm (Festool Group GmbH & Co. KG, Wendlingen, Germany).

Surface treatments (Table 1) were applied as recommended by producers in the technical documents. The coating films were created with one (I), two (II), and three (III) layers of airless spraying. Applied surface coatings are:

- Polyurethane—solvent-based finish,
- Polyurethane-acrylate—water-based finish.

The test samples were conditioned in horizontal position at 23 ± 2 °C and 50 ± 5% relative humidity for 5 days.

### 2.2. Coating Thickness

The dry film thickness of the coatings was determined using a non-destructive ultrasonic thickness gauge (PosiTector^®^ 200, DeFelsko Corporation, Ogdensburg, NY, USA). Measurements were performed at ten randomly selected locations on each specimen to ensure representative values.

### 2.3. Exposure in Natural Ageing

Natural ageing was carried out under indoor conditions with exposure to natural daylight. The specimens were placed behind a glass window consisting of thermal-insulation double glazing (U-factor 1.1 W·m^−2^·K^−1^), oriented to the west, in order to allow exposure to solar radiation while minimising the influence of external climatic factors (e.g., precipitation and direct weathering). The exposure was conducted at the Technical University in Zvolen (GPS: 48.572024, 19.118499; altitude 283 m above sea level). During the exposure period, the indoor temperature was maintained within the range of 20–25 °C, and the relative humidity fluctuated between 50% and 55%, simulating standard interior conditions.

Colour measurements were performed after 30, 150, and 300 days of exposure to natural light.

During the monitoring period, the cumulative sunshine duration reached 2000 h, corresponding to an average of approximately 6 h 40 min of sunshine per day. The cumulative sunshine hours reached 200 h more than the average. The maximum UV index recorded at the location was 6.5, corresponding to a high level of UV radiation.

### 2.4. Evaluation of Colour of Surface Treatment 

The colour changes on sample surfaces were visualised and quantified using the CIELAB colour space. The CIELAB system is a three-dimensional model consisting of three axes: the L^*^-axis represents lightness (L^*^ = 100 corresponds to white, L^*^ = 0 to black), while chromaticity is described in the ab plane—with a^*^ ranging from green (−) to red (+) and b^*^ from blue (−) to yellow (+).

The measurements were taken at 10 spots on each sample (both on false heartwood and mature wood). Colour measurements were carried out with a spectrophotometer (Spectro-Guide 45/0 Gloss, BYK-GARDNER GmbH, Geretsried, Germany). The colour coordinates were measured based on a spherical measurement geometry with a 45°/0° measurement geometry, with a test window diameter of 11 mm.

The coordinates L^*^, a^*^, and b^*^ were measured before exposure and at specified time intervals after exposure to natural ageing indoors. From the averaged values of measured coordinates before and after exposure, the differences between the individual coordinates ΔL^*^, Δa^*^, and Δb^*^ were calculated using the following equations:

∆L^*^ = L^*^_2_ − L^*^_1_ (1)

∆a^*^ = a^*^_2_ − a^*^_1_ (2)

∆b^*^ = b^*^_2_ − b^*^_1_ (3)

where index ‘1’ represents the colour coordinate value of the referential sample before exposition; index ‘2’ is the value of the same coordinate after exposition.

The total colour difference ∆E^*^_ab_ (−) was subsequently determined using the following equation [33]:

(4) $$∆{E^{*}}_{\mathrm{ab}}=\sqrt{{∆L}^{*2}+{∆a}^{*2}+{∆b}^{*2}}$$

The colour difference in the surfaces after natural ageing was evaluated based on Table 2.

### 2.5. Evaluation of Gloss of Surface Treatment

On test specimens, having a different number of layers (I, II, III), the gloss of the surface was measured perpendicular to wood fibres by the standard STN EN 13722 [35]. To measure the gloss, a spectrophotometer (Spectro-Guide 45/0 Gloss, BYK-GARDNER GmbH, Geretsried, Germany) was used. This type of spectrophotometer is used to measure the gloss at an angle of 60° with a test window of 5 mm *×* 10 mm. This device is equipped with an improved technical specification for an angle of 60°, also in the degree of gloss, matt (0–100). On each test specimen, three measurements of gloss G (GU) were done in a direction perpendicular to the fibres.

### 2.6. The Statistical Evaluation

The MS Excel and statistical software STATISTICA 14 were used to analyse and present the collected data on colour difference and gloss parameters. Descriptive statistics deal with basic statistical characteristics—arithmetic mean and standard deviation—and an analysis of variance (ANOVA) at a 0.05 significance level. In the statistical processing of data, a four-way Analysis of variance was used to examine the effects of four independent factors—zone, surface treatment, time, and number of layers on the response variables—colour difference and gloss. This approach allowed us to assess both main effects and interaction effects among the factors. To complement the ANOVA results, the confidence intervals 95% were calculated for the mean values of colour difference and gloss for each group. All analyses were performed assuming normality and homogeneity of variances.

### 2.7. Evaluation of Resistance to Mould Fungi

Resistance to mould fungi was determined using a method based on the standard STN EN 15457 [36]. Coated and uncoated (control) wood specimens were exposed to a mixed spore suspension of pure cultures of *Aspergillus niger* Tiegh—and *Penicillium brevicompactum* Dierckx. The fungal strains were obtained from the Mycological Laboratory of the Faculty of Wood Sciences and Technology, Technical University in Zvolen (Slovakia). The fungal suspension was applied to the surface of the test specimens by spraying. Each specimen was individually placed on the surface of Czapek–Dox agar medium in Petri dishes (100 mm diameter, 15 mm height). Incubation was carried out at 27 ± 1 °C and 65% relative humidity in the climatic chamber (Memmert, Schwabach, Germany).

The growth activity of mould (GAM) was evaluated after 7, 14, and 21 days of exposure in accordance with the standard [36]. Coated and uncoated (control) wood specimens were exposed to conditions (24 ± 2 °C; 50–95% relative humidity). The assessment was performed visually with the naked eye and using a stereomicroscope (10× magnification). The degree of surface colonisation was classified on a 0–4 rating scale according to the percentage of the specimen’s upper surface covered by mould growth: 0—no visible growth, 1—growth up to 10% of the surface area, 2—growth up to 30% of the surface area, 3—growth up to 50% of the surface area, and 4—growth exceeding 50% of the surface area. The evaluation was performed on the upper surface of each specimen.

## 3. Results and Discussion

### 3.1. The Coating Thickness

The dry film thickness for polyurethane coating and polyurethane-acrylate coating, created with one (I), two (II), and three (III) layers, is given in Table 3. With the increasing number of layers, the coating film thickness did not increase proportionally. The thickness of the coating films of both surface treatments was almost the same.

Van Acker et al. [37] state that continuous coating films are usually achieved at dry film thicknesses between 20 μm and 30 μm. Both finishes achieve this condition already with one layer. Continuous coating films in high thickness can very well protect the wood against external influences such as chemicals, moisture, abrasion, and dirt [37]. Similarly, in our research, we assumed that the surface treatment would require two to three layers to achieve the required protective properties.

### 3.2. The Colour of Surface Treatment After the Natural Ageing

**Figure 1:**
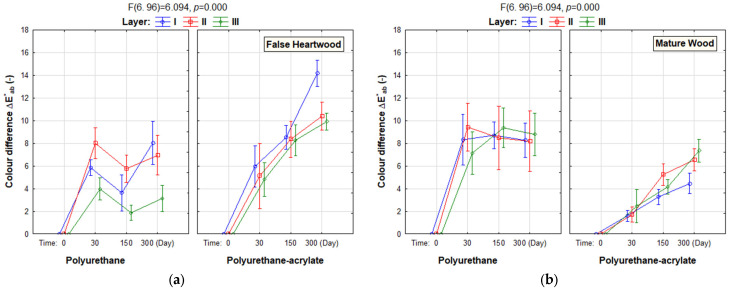
The colour difference ∆E^*^_ab_ on false heartwood (**a**) and mature wood (**b**), on polyurethane and polyurethane-acrylate finishes with three coating film thicknesses after natural ageing in the interior. (NOTE: The F-statistic is 6.094 (with the corresponding degrees of freedom of 6.96); the *p*-value is less than 0.001—a highly statistically significant difference).

Figure 1a shows that the largest colour difference ∆E^*^_ab_ on false heartwood, on the surface treatment polyurethane, occurred after 30 days. The biggest colour difference was on two layers (the difference is evaluated as another colour), smaller on one layer (the difference is evaluated as another colour), and the smallest on three layers (a significant colour difference that is rarely accepted).

After 150 days, the colour difference on all surfaces was significantly reduced. On the surfaces, the classification was: on one layer “significant colour difference that is rarely accepted”, on two layers “the difference evaluated as another colour”, on three layers “perceived colour difference that is certainly seen”.

After 300 days, the colour difference increased on all surfaces; on the surfaces with one and two layers, “the difference is evaluated as another colour”. On the surface with three layers, the colour difference was “perceived colour difference that is certainly seen”.

The largest colour difference ∆E^*^_ab_ (the difference is evaluated as another colour), on false heartwood on the polyurethane-acrylate surface treatment, occurred after 300 days on all layers (I, II, III). The largest colour difference ∆E^*^_ab_ = 14 was on one layer. Colour difference showed an increasing tendency with time for all numbers of layers. After 30 days, the classification was “significant colour difference that is seldom accepted”. After 150 days, the colour difference ∆E*ab was about 8 (the difference is evaluated as another colour). After 300 days, the colour difference ∆E^*^_ab_ was around 10 (two and three layers) and ∆E^*^_ab_ = 14 on one layer; the classification was “the difference is evaluated as another colour”.

The results of the mature wood on the polyurethane surface treatment are presented in Figure 1b. The significant colour difference ∆E*ab on mature wood, on the polyurethane surface treatment, occurred after 30 days on all layers (I, II, and III). Colour difference on all layers reached “the difference is evaluated as another colour”. After 150 and 300 days, all layers showed comparable colour differences, classified “the difference is evaluated as another colour”.

The largest colour difference ∆E^*^_ab_ on mature wood, on the polyurethane-acrylate surface treatment, occurred after 300 days on all layers (I, II, and III). After 30 days, the colour differences on the surfaces with one and two layers were classified “observable colour difference that is barely seen”, and with three layers, classified “perceived colour difference that is certainly seen”. After 150 days, the surfaces with one layer, the colour difference was classified “perceived colour difference that is certainly seen”, with two layers “the difference is evaluated as another colour”, and with three layers “significant colour difference that is seldom accepted”.

The results of the colour difference ∆E^*^_ab_ were from the point of the wood zones. The false heartwood with a solvent-based finish—polyurethane with three layers had a smaller colour difference than mature wood. False heartwood with a water-based finish—polyurethane-acrylate with each number of layers showed a larger colour difference than mature wood. Colour variations on false heartwood with a water-based polyurethane-acrylate finish continuously increased over time more than on mature wood.

The results of the false heartwood with a solvent-based finish—polyurethane surface treatment are presented in Figure 1. The course of colour differences over time on false heartwood with a solvent-based finish—polyurethane had the same course on all layers; only on three layers, the colour differences were significantly smaller than on one and two layers after 300 days. A significant difference in colour difference ∆E^*^_ab_ was seen on false heartwood with a water-based finish—polyurethane-acrylate with two and three layers, after 300 days, it was significantly smaller than on one layer. A significant difference in colour difference ∆E^*^_ab_ was seen on mature wood with a water-based finish—polyurethane-acrylate with two and three layers, after 300 days, it was significantly higher than on one layer.

**Figure 2:**
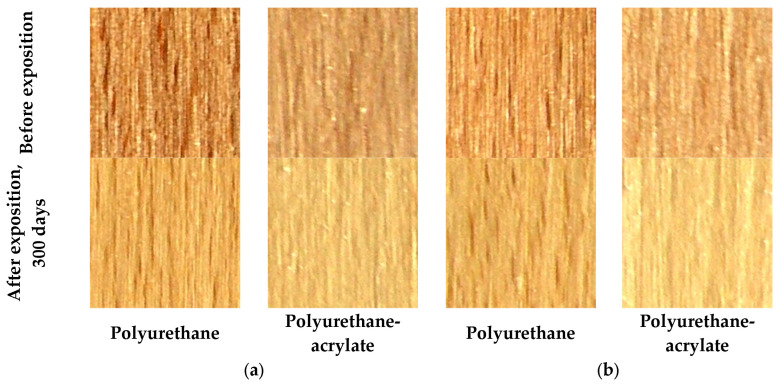
The colour difference ∆E^*^_ab_ on false heartwood (**a**) and mature wood (**b**), on polyurethane and polyurethane-acrylate finishes with three layers before and after natural ageing in an interior for 300 days.

The positive effect of coating thickness on reducing the colour difference was evident for both coatings after 300 days, only on the false heartwood (Figure 2). On mature wood, the water-based finish—polyurethane-acrylate coating had a greater colour difference on the thicker coating film. One factor that contributed to the change in surface colour was the coating film, and the other factor was the colour of the wood.

Forsthuber et al. [38] concluded that the contrast of earlywood and latewood of Siberian larch (*Larix sibirica* L.), when using two different coating films based on waterborne acrylate dispersion, was different. This was also in agreement with the visible light transmittance of the coatings measured between 360 and 800 nm, where one coating showed a visible light transmittance of 86.7% and the second coating a visible transmittance of 76.8%. The differences could also be affected by the differences in dry film thickness of the coating systems used.

Forsthuber et al. [38] stated that the discoloration after exposure to xenon was fast, up to 3 h of exposure. Afterwards, the colour changes were much slower. It can be confirmed that the solvent-based finish—polyurethane on mature wood behaved similarly. A different course of colour difference during exposure was seen on the false heartwood.

From the point of view of surface finishes, other authors stated that the accelerated ageing process with simulated indoor conditions induced more significant discolouration of wood surfaces coated with solvent-based polyurethane lacquers if compared with water-based coating systems [12,13]. This statement was also confirmed by our results on mature wood after 300 days of natural ageing indoors, but on false heartwood, the results were the opposite. Water-based finish—polyurethane-acrylate was less stable than the solvent-based finish—polyurethane.

Torcătoru and Timar [39] compared the light-induced colour changes in uncoated and coated wood surfaces of European maple (*Acer pseudoplatanus* L.). Exposure of uncoated maple wood resulted in a total colour difference of 10.83, and this was only slightly reduced by coating. Exposure through the glass slide reduced the colour difference in the uncoated and coated wood surfaces by 37–43%. This statement can be compared with our results of the influence of the number of coatings on the colour difference after the ageing process.

It can be confirmed that after the exposure of 300 days, the solvent-based finish—polyurethane in two layers on false heartwood showed reduced colour difference by 12.5% if compared with one layer. The surface with three layers showed a reduced colour difference by 62.5%. The water-based finish—polyurethane-acrylate in two or three layers on mature wood showed reduced colour difference by 26.5–30% if compared with one layer.

Two- and three-layer water-based finish—polyurethane-acrylate on mature wood showed a reduced colour difference by 14.5–15.5% if compared with one layer. If the impact of surface colour change is evaluated from the perspective of wood colour, it must be taken into account that wood is a non-homogeneous material with differently coloured parts. Dudiak et al. [40] concluded that under the influence of UV radiation, beech wood with false heartwood undergoes intense yellowing and darkening due to photooxidation of native lignin.

For the solvent-based polyurethane finish on false heartwood, the colour difference was very large at 30 days and then decreased significantly at 150 days. Dudiak et al. [40] reached the same conclusion. The course of the colour difference on the natural false heartwood was identical to that recorded on the polyurethane surface treatment. Based on this, it can be assumed that the course of the colour difference in this surface treatment was mainly influenced by the substrate. The resulting surface colour is determined by the interaction of the colour of the wood with the transparent coating. In the process of natural ageing, the colour of the wood is changing, and the colour of the coating is changing as well.

The natural ageing of wood is an irreversible, time-occurring complex phenomenon, which affects both the surface of wood and the surface treatment. During the natural or accelerated ageing of wood, lignin was mainly degraded, especially in the early stages of the process, and a linear correlation was stated between the changes in lignin and the colour changes in the wood [41].

Kúdela et al. [42] proved that in the case of spruce wood, surface-treated with lacquer based on acryl-polyurethane dispersions without lignin stabiliser, the discolouration was the same as that of the substrate, and this was true throughout the whole ageing process. In all the solid coating films, there are significant ageing process-related changes in colour difference.

Photo-discoloration of clear-coated wood may be caused by the yellowing of both clear coating film and underlying wood, or by either of them [9]. From the results, it is seen that different coloured zones of the wood (mature wood, false heartwood) reacted differently to natural light and changed their colour differently. Authors of [29] monitored the change in colour due to the chemical composition of the beech wood. They found that the relative content of lignin and hemicelluloses was highest in the false heartwood and the lowest in the sapwood. On the contrary, the relative content of holocellulose and cellulose was the lowest in the false heartwood and the highest in the sapwood. The relative content of extractive substances was higher in the sapwood than in the false heartwood.

Different types of wood with the same type of surface treatment behaved differently as well. This was also confirmed by the research of [43]. If wood of two different wood species was coated with two different types of waxes, the course of the colour difference for individual wood species was different. The colour differences under the influence of natural light, on two different surface treatments on the same type of wood with different zones, were also different. Our results confirmed that the specific colour difference resulted from the interaction of the coating films with various zones in wood. The zones are of different chemical composition, and therefore, the wood zones are coloured differently.

Authors of [29] concluded that the colour differences in mature wood and false heartwood result from chemical changes in beechwood during tree growth. The browning of mature wood and false heartwood was attributed to lignification of the cell walls of mature wood and false heartwood; in the case of false heartwood, to a decrease in the cellulose content and the formation of polyphenolic compounds (cause a red-brown colour).

Authors of [44] investigated the spectral changes in beech wood during natural indoor ageing. The differently coloured zones of the wood (mature wood, false heartwood) reacted differently to natural light and changed their colour differently. These spectral changes reflect the degradation of the basic structural components of wood, especially lignin and hemicelluloses, as a result of natural ageing under light exposure.

The chemical changes in the wood caused these zones of the wood to have different photostability. This was reflected in the surface treatments, where the course of the colour differences was different. Likewise, the solvent-based finish—polyurethane showed a different course of colour differences on the false heartwood from the course on mature wood.

### 3.3. The Gloss of Surface Treatment After the Natural Ageing

**Figure 3:**
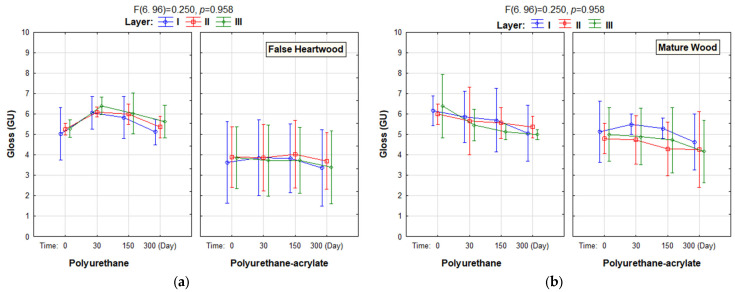
The gloss (GU) on false heartwood (**a**) and mature wood (**b**), on polyurethane and polyurethane-acrylate finishes with three coating film thicknesses after natural ageing in the interior. (NOTE: The F-statistic (with the corresponding degrees of freedom 6.96) is 0.250, and a large *p*-value (*p* > 0.05) indicates that the differences are not statistically significant).

The results of the experiment on the false heartwood with a solvent-based finish—polyurethane surface treatment are listed in Figure 3a. The gloss of the surface of false heartwood with a solvent-based finish—polyurethane did not change significantly with the number of layers. The statistically significant change after 30 days was on the surface with two and three coats. The gloss has increased slightly, but it was still in the matte range. After 150 days, the gloss had decreased slightly, and after 300 days, it had decreased to the original value, but this change was not statistically significant.

The gloss of the surface of false heartwood with a water-based finish—polyurethane-acrylate did not change significantly with the number of layers, nor with the number of days of exposure.

The results of the mature wood with a solvent-based finish—polyurethane surface treatment are presented in Figure 3b. The gloss of the surface of mature wood with a solvent-based finish—polyurethane did not change significantly with the number of layers. The change in gloss over exposure time was also statistically insignificant, with gloss only slightly decreasing on the surface with all numbers of layers.

There was no statistically significant difference in gloss on the surface of mature wood with water-based finish—polyurethane-acrylate from the point of view of the number of layers, and also from the point of view of exposure time.

In the case of matte finishes, it has not been confirmed that the gloss or matte increases with the increasing number of coats. However, in the paper by study [45], which investigated polyurethane semi-matte surface treatments, the effect of the number of layers on gloss was evident. It was more pronounced on water-based than on solvent-based surface treatments. It also did not change significantly, even with natural ageing in the interior after 300 days. But in the studies by [9,46], that in general, the surface gloss is reduced with ageing. In the paper [6], the gloss values of coated wood specimens after 3 months of natural weathering were slightly low, and after 6 months of natural weathering, the decrease reached a considerable level. In the paper by [47], the results obtained for the colour and gloss demonstrate that the coating system had a high resistance against photodegradation after artificial ageing. Under the dry mode (UV radiation without rainfall simulation), no obvious changes to the coating occurred in colour and gloss.

A statistically significant change in gloss was recorded only on the false core on solvent-based finish—polyurethane with three layers after 30 days, the level of gloss has increased. But even this change was not observable with the naked eye, because the measured gloss values were still in the range of a matte surface.

Gloss of coating films (solvent-based finish or water-based finish) with 1, 2 or 3 layers was ranked to the degree of gloss “matte” (to 10 GU) to “deep matt” (to 5 GU). A difference in the degree of gloss may occur in the case of the surface with two or three layers reaching the upper limit of the degree of gloss. By increasing the number of layers, we reach a higher degree of gloss or a deeper matte.

In practice, the trend is not to increase the number of layers on all surfaces of one furniture piece. As reported by studies [3,4,5], increasing the number of layers, VOCs in the atmosphere are increasing, and the price of furniture is increasing. Therefore, a higher number of layers is applied only onto the surfaces that are mechanically stressed. These areas may be glossier or duller than the other areas of the same furniture piece. But in the paper by [5] little correlation between the gloss and the number of coatings; increasing the number of coatings did not significantly improve gloss.

### 3.4. The Influence of Layers and Other Factors

Calculated values of colour difference ∆E^*^_ab_ and gloss G were evaluated by four-factor analysis of variance in the Statistica programme (Table 4).

We evaluated the effect of wood zone (false heartwood or mature wood—2 levels of factor 1), the different number of layers (3 levels of factor 2), the type of coating material (2 levels of factor 3), and the effect of the length of natural light on the surface of the specimens (3 levels of factor 4). It was confirmed that the observed factors and interactions were statistically highly significant for the colour difference ∆E^*^_ab_.

For the effect on surface gloss, it was confirmed that only the following observed factors and interactions were statistically highly significant: Zone, Surface treatment, Time, Zone–Surface treatment, and Zone–Time. The following interactions were statistically significant: Zone–Layer and Zone–Surface treatment–Time (Table 4).

### 3.5. The Resistance to Mould Fungi

Untreated wood surfaces are readily colonised by various microorganisms, particularly microscopic fungi (moulds), when exposed to favourable environmental conditions. One of the principal functions of surface coatings is therefore to enhance the resistance of wood against environmental factors and biodeterioration [11,48,49,50,51]. Conversely, microbial colonisation may induce structural changes in the coating film, leading to deterioration of its mechanical properties and protective performance [52]. No difference in mould growth was observed between the zone of the false heartwood and mature beech wood; therefore, subsequent analyses focus on coating type and number of layers.

**Figure 4:**
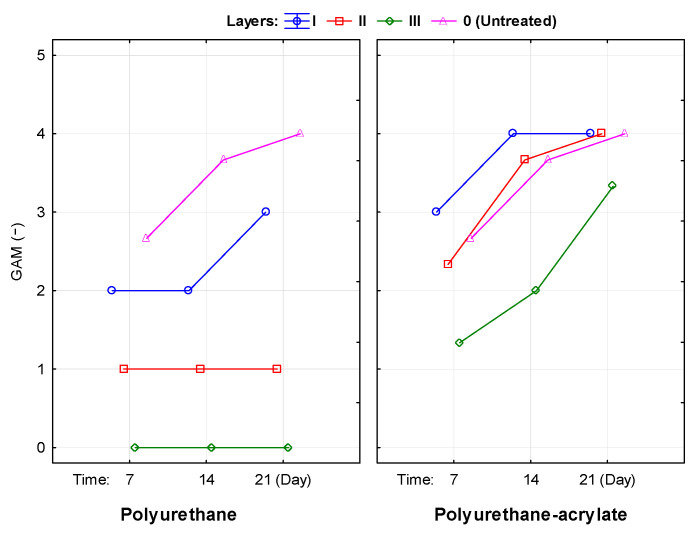
Development of mould growth activity (GAM) during 21-day exposure on untreated and coated beech wood with different layer thicknesses.

**Figure 5:**
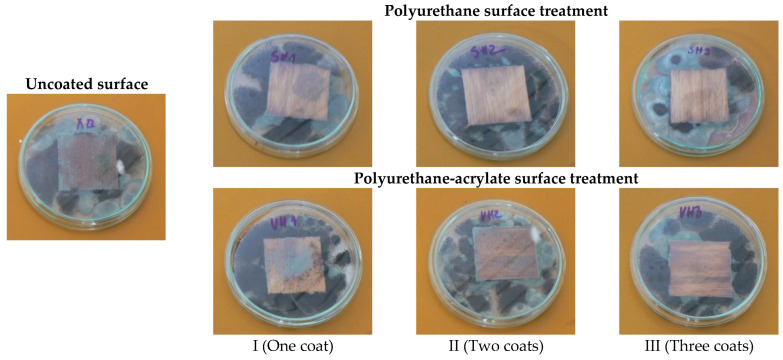
The GAM on the coated and uncoated surfaces after 14 days of exposure.

The results of the visual assessment of mould resistance for uncoated and coated specimens are presented in Figure 4 and Figure 5. The degree of mould growth was primarily influenced by the coating formulation, the number of applied layers, and the exposure duration. As expected, the untreated wood exhibited no resistance to mould colonisation.

For the solvent-based polyurethane coating, complete inhibition of mould growth (GAM = 0) was observed only for the three-layer system throughout the entire exposure period (7 and 21 days), whereas the one- and two-layer systems exhibited measurable mould growth.

In contrast, the water-based polyurethane–acrylate coating showed higher susceptibility to mould development. Mould growth was comparable for one- and two-layer applications, while a more pronounced inhibitory effect was observed for the three-layer system. Overall, increasing coating thickness reduced mould growth; however, even the three-layer polyurethane–acrylate system exhibited reduced but still progressive growth over time (GAM = 1.5 after 7 days and 3.0 after 21 days for thinner coatings).

Overall, an increase in the number of applied layers resulted in reduced mould growth, indicating that film thickness plays an important role in enhancing mould resistance. Comparable results were reported by [53], who observed reduced mould colonisation following treatment with polyurethane resins. Study [52] reported higher mould growth on commercial polyurethane–acrylate coatings compared with conventional polyurethane systems, suggesting that insufficient particle coalescence may reduce coating integrity and thereby decrease biological resistance.

Further studies have shown that intrinsic differences in coating systems, such as film structure and surface properties, are associated with distinct patterns of fungal development on coated wood [16]. In addition, evaluations of antifungal performance across various commercial coatings indicate that coating composition and adhesion properties significantly influence biological resistance under laboratory conditions [14,54]. These findings suggest that mould resistance is governed not only by coating thickness, but also by the physicochemical characteristics of the coating film and its interaction with moisture.

## 4. Conclusions

1. The results demonstrated that the number of applied layers of polyurethane solvent-based and polyurethane-acrylate water-based finishes and duration of exposure significantly influence both colour stability and susceptibility to mould growth on beech wood surfaces with zones of false heartwood and mature wood.
2. A significant colour change in the solvent-based polyurethane finish on both mature wood and false heartwood was observed after 30 days of natural ageing, while the water-based polyurethane-acrylate finish showed a progressive increase in colour difference over time.
3. After 300 days, the importance of applying a higher number of coating layers became evident, particularly on false heartwood, where three layers resulted in improved colour stability for both surface finishes. In the case of mature wood, this effect was confirmed only for the polyurethane-acrylate water-based finish, as the number of coating layers increased, the colour difference.
4. Natural ageing in the interior environment did not affect the gloss of matte surfaces for either surface finish, regardless of wood zone.
5. Untreated beech wood proved to be highly susceptible to mould growth under favourable conditions, highlighting its potential environmental risk. The extent of mould development was primarily governed by the type of surface finish, number of layers, and exposure time, with higher mould activity observed on polyurethane-acrylate surfaces compared to polyurethane ones.
6. Increasing the number of coating layers effectively reduced mould growth for both surface finishes.

## Figures and Tables

**Table 1:** Characteristics of Coating Materials and Their Applications.

Surface Treatment	Solvent-Based Finish	Water-Based Finish
Surface coatings	Polyurethane	Polyurethane-acrylate
Components	Two-component	Two-component
Film former	Acrylate resin	Polyurethane-acrylic- copolymer dispersion basis
Gloss	Matte	Matte
Mixture ratio (%)	10:2	100:7
Viscosity 4 mm-cup (s)	30	200 ± 10
Drying parameters	4 to 12 h at 23 °C, RH 50%	3 h at 23 °C, RH 50%
Spread rate (mL·m^−2^)	100 to 150	100 to 120
Application	Airless	Airless
Spray nozzle (ø mm)	0.23 to 0.28	0.23 to 0.33
Hardener	Solvent polyisocyanate	Solution of aliphatic polyisocyanate
Sand grit	P150 to P180	P150 to P180
Intermediate grinding	P240 to P320	P240 to P320

NOTE: RH—Relative humidity. The data were obtained from and processed according to the technical data sheets [31,32].

**Table 2:** Colour change assessment [34].

Colour Difference		Colour Change Classification
1	0.5 < ΔE^*^_ab_	No to nearly no colour difference
2	0.5 < ΔE^*^_ab_ < 1	The difference can be perceptible for the practiced eye
3	1 < ΔE^*^_ab_ < 2	An observable colour difference that is barely seen
4	2 < ΔE^*^_ab_< 4	Perceived colour difference that is certainly seen
5	4 < ΔE^*^_ab_< 5	Significant colour difference that is seldom accepted
6	ΔE^*^_ab_ > 5	The difference is evaluated as another colour

**Table 3:** Dry film thickness created with one (I), two (II), and three (III) layers.

Surface Treatments	Dry Film Thickness [µm]		
	I	II	III
Polyurethane	55 (30–60)	71 (61–90)	95 (91–120)
Polyurethane-acrylate	53 (30–60)	74 (61–90)	100 (91–120)

Note: The values represent the arithmetic mean of ten measurements. The minimum and maximum values are indicated in parentheses.

**Table 4:** The Four-Factor Analysis of Variance for the Colour Difference and Gloss.

Factor	Level of Significance—*p*	
	Colour ∆E^*^_ab_ (−)	Gloss G (GU)
Zone	0.000	0.000
Layer	0.000	0.693
Surface treatment	0.000	0.000
Time	0.000	0.000
Zone–Layer	0.000	0.021
Zone–Surface treatment	0.000	0.000
Layer–Surface treatment	0.000	0.309
Zone–Time	0.000	0.001
Layer–Time	0.000	0.714
Surface treatment–Time	0.000	0.866
Zone–Layer–Surface treatment	0.000	0.201
Zone–Layer–Time	0.000	0.960
Zone–Surface treatment–Time	0.000	0.010
Layer–Surface treatment–Time	0.000	0.989
Zone–Layer–Surface treatment–Time	0.000	0.958

## Data Availability

The data that support the findings of this study are available on request from the corresponding author.
